# The effect of small changes in rate of force development on muscle fascicle velocity and motor unit discharge behaviour

**DOI:** 10.1007/s00421-022-04905-7

**Published:** 2022-02-10

**Authors:** Jeroen Aeles, M. Bellett, G. A. Lichtwark, A. G. Cresswell

**Affiliations:** 1 School of Human Movement and Nutrition Sciences, The University of Queensland, Brisbane, QLD Australia; 2 Laboratory “Movement, Interactions, Performance” (EA 4334), University of Nantes, Nantes, France

**Keywords:** Muscle fascicles, Contraction dynamics, Ultrasound, Fine-wire, Motor unit

## Abstract

When rate of force development is increased, neural drive increases. There is presently no accepted explanation for this effect. We propose and experimentally test the theory that a small increase in rate of force development increases medial gastrocnemius fascicle shortening velocity, reducing the muscle’s force-generating capacity, leading to active motor units being recruited at lower forces and with increased discharge frequencies. Participants produced plantar flexion torques at three different rates of force development (slow: 2% MVC/s, medium: 10% MVC/s, fast: 20% MVC/s). Ultrasound imaging showed that increased rate of force development was related to higher fascicle shortening velocity (0.4 ± 0.2 mm/s, 2.0 ± 0.9 mm/s, 4.1 ± 1.9 mm/s in slow, medium, fast, respectively). In separate experiments, medial gastrocnemius motor unit recruitment thresholds and discharge frequencies were measured using fine-wire electromyography (EMG), together with surface EMG. Recruitment thresholds were lower in the fast (12.8 ± 9.2% MVC) and medium (14.5 ± 9.9% MVC) conditions compared to the slow (18.2 ± 8.9% MVC) condition. The initial discharge frequency was lower in the slow (5.8 ± 3.1 Hz) than the fast (6.7 ± 1.4 Hz), but not than the medium (6.4 ± 2.4 Hz) condition. The surface EMG was greater in the fast (mean RMS: 0.029 ± 0.017 mV) compared to the slow condition (0.019 ± 0.013 mV). We propose that the increase in muscle fascicle shortening velocity reduces the force-generating capacity of the muscle, therefore requiring greater neural drive to generate the same forces.

**Supplementary Information:**

The online version contains supplementary material available at 10.1007/s00421-022-04905-7.

## Introduction

Precise control of muscle force output is a critical factor in enabling the broad and complicated range of movements that humans can perform. Adjusting the neural drive, i.e. the sum of all efferent discharges, to a muscle is required to rapidly modify a muscle’s force output. The neural drive can be increased by either increasing the discharge frequency of the active motor units or by recruiting more motor units or both. If the recruitment threshold of the motor units is lowered, then more motor units will be recruited for a given force output. Therefore, increasing the neural drive for a given force output by recruiting more motor units can also be achieved indirectly by recruiting motor units earlier, i.e. by lowering their recruitment thresholds. However, the muscle must also work within its mechanical constraints. These constraints include factors such as muscle length (Gordon et al. 1966) and velocity (Hill 1938), which dictate the force output of the muscle for a given activation level. While the motor unit discharge behaviour and internal dynamics of the muscle, i.e. the muscle fascicle velocity, have been separately studied, their interplay is not well understood and is critical in understanding the control of human movement.

There is a well-documented link between the rate of force development and muscle activation. During voluntary contractions to a set force, but with an increasing rate of force development, whole muscle activation measured by integrated electromyography (EMG) increases with the increase in force rate (Bigland and Lippold 1954; Sale 1987; MacIntosh et al. 1999; Farina et al. 2004). While some discrepancies on this topic exist (Nelson et al. 1973; Barnes 1980; Komi 1973; MacIntosh et al. 1999), they can mainly be explained by how force or rate was controlled. Accounting for these methodological discrepancies, findings consistently demonstrate that voluntary control of muscle activation must be adjusted to meet the changing mechanical demands of the task.

Increasing the activation of a muscle during faster force ramps can be achieved by increasing the number of active motor units. This has been observed in several different experiments, as well as the finding that motor units recorded are often recruited at progressively lower force levels (i.e. lower recruitment thresholds) as the rate of force development increases (Seyffarth 1940; Tanji and Kato 1973; Freund et al 1975; Budingen and Freud 1976; Desmedt and Godaux 1977a). In addition, the frequency at which the active motor units fire also appears to increase with increasing rate of force development, with this being studied in rather slow versus extremely fast or ballistic contractions (Grimby and Hannerz 1977; Desmedt and Godaux 1977a, b, 1979; Duchateaux and Baudry 2014). Each of these changes to the motor unit behaviour are to meet the demand for increased neural drive with an increase in the rate of force development requirement.

While there appears to be a set relationship between the rate of force development and motor unit discharge behaviour, consensus on an explanation driving this behaviour is lacking. Proposed explanations include the de-recruitment of motor units innervating slow fibres (Barnes 1980; Sale 1987) and co-contraction of agonist/antagonist muscles (Barnes 1980). However, even though these theories have been proposed, no experimental supporting evidence has been found. As it stands, there is currently no experimentally confirmed theory that explains why an increase in rate of force development is paired with an increased neural drive and activation to the muscle.

Here we propose that the muscle fascicle dynamics during different contraction rates play a role in the increased neural drive response. That is, according to the well-accepted force–velocity relationship of muscle, described by Hill in 1938, the force capacity of a muscle is significantly reduced when its shortening velocity is increased (Hill 1938). Even during isometric contractions, muscle fascicles shorten because of in-series compliance of their tendons (Griffiths 1984, 1987; Mayfield et al. 2015). Because fascicle shortening amplitude is directly related to force, higher rates of force development should require higher fascicle shortening velocity, which would inevitably reduce the force-generating capacity of active fascicles. We propose that this increase in fascicle shortening velocity and drop in force-generating capacity drives the changes observed in motor unit behaviour when rate of force development is increased.

The aim of this study was to provide evidence for why there is a need for increased neural drive, even with small increases in rate of force development during submaximal isometric contractions. In a first experiment, we provide evidence that with small increases in rate of force development, the shortening velocity of the medial gastrocnemius (MG) muscle fascicles is invariably increased. In a separate experiment, we then tested the effect of these small increases in rate of force development on motor unit behaviour, under similar conditions as the first experiment. We hypothesised that with increasing rate of force development, we would see increased fascicle shortening velocity with concomitant lowering of motor unit recruitment thresholds and increases in motor unit discharge frequencies, as well as an overall increase in myoelectric activity, reflecting additional motor unit recruitment, to compensate for the drop in the muscle’s force-generating capacity.

## Methods

Data were collected in two experiments from two similar and overlapping participant groups. Ultrasound data (exp. 1) were obtained from eight participants (seven men and one woman, height: 179 ± 4 cm, mass: 80 ± 13 kg, age: 26 ± 4 years). After confirming the premise set for experiment 1, we then collected motor unit data (exp. 2) obtained from nine participants (six men and three women, height: 178 ± 9 cm, mass: 76 ± 14 kg, age: 33 ± 11 years), of which three (two men, one woman) were tested on two separate occasions to increase the number of motor units available for analysis. Two participants performed both experiment 1 (ultrasound) and experiment 2 (motor unit). All participants were healthy adults and provided written informed consent. The study was approved by The University of Queensland local Human Research Ethics Committee (file number: 2011001398) and the experiments were performed in accordance with the Declaration of Helsinki.

### Protocol and data collection—experiment 1

**Fig. 1:**
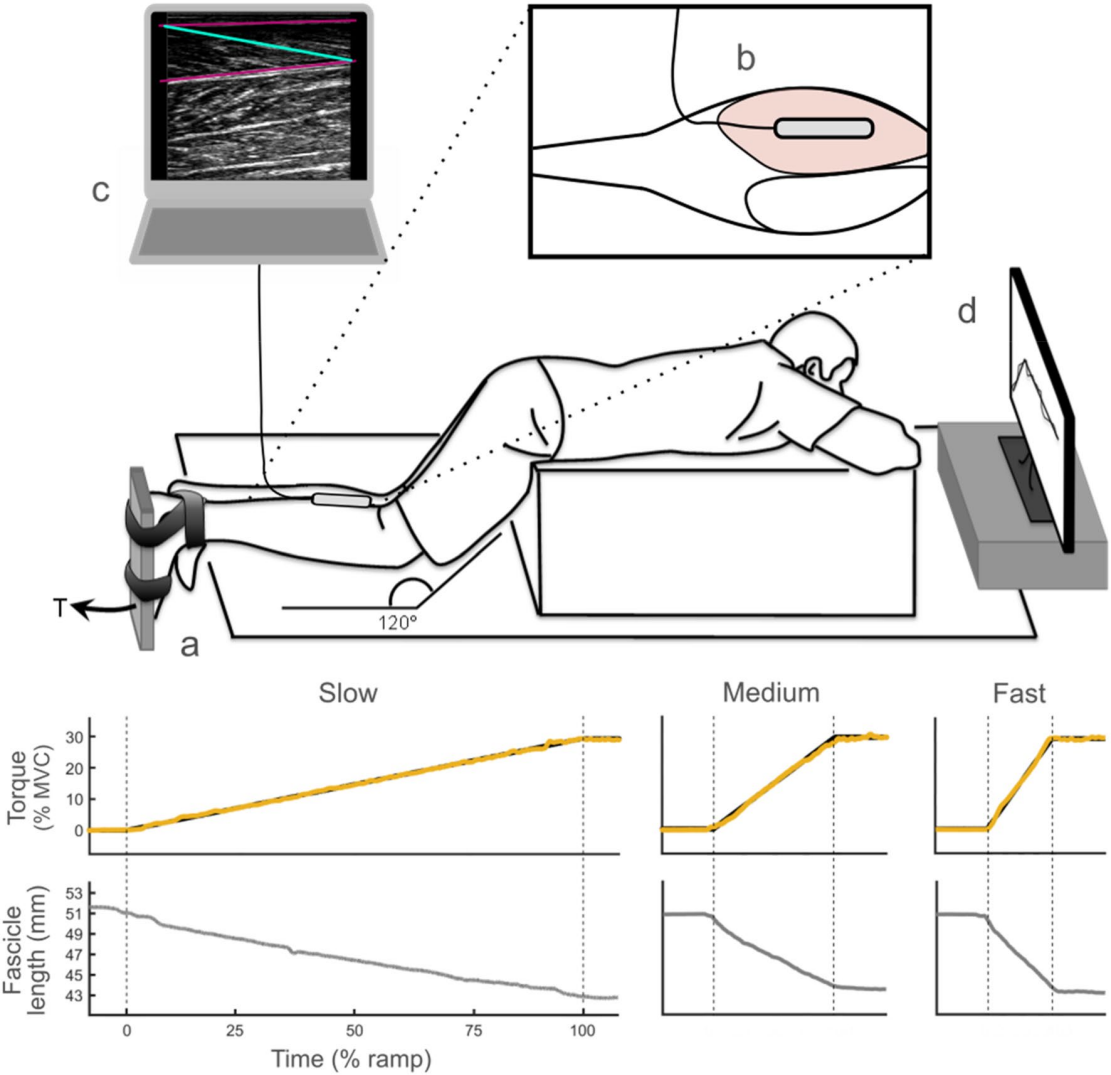
Top panel: overview of the setup for experiment 1 with a zoomed-in top view. The participants performed a plantar flexion against a rigid foot plate that measured plantar flexion torque (**a**). Muscle fascicle dynamics were measured from the medial gastrocnemius (MG) using ultrasound imaging (**b**). The deep and superficial aponeuroses of the muscle (**c**, pink lines) and the mean muscle fascicle orientation (**c**, green line) were tracked to calculate fascicle length. Visual feedback of the required ramp and plantar flexion target torque was displayed on a monitor in front of the participants (**d**). Bottom panel: example data from one participant for all three conditions (slow, medium, fast). Target torque (black) and the participant’s torque (orange) on top, the fascicle length below. 1-s before and after each ramp is shown for all data. The vertical dashed lines mark the start and end of each ramp

Participants were positioned prone on an examination table with their trunk raised and supported by a padded wooden box to position their knee joints at 120°—internal angle (Fig. 1). Their preferred foot was firmly secured with strapping to a rigid plate that was connected to a torque transducer (Maywood Instruments, Basingstoke, UK). The combined foot and plate were positioned with the ankle at 90°, measured as the angle between the tibia and sole of the foot. After a brief warm-up, consisting of isometric, submaximal plantar flexion contractions, each participant was asked to perform three plantar flexion maximal voluntary contractions (MVCs) with at least 60 s rest between each MVC. The highest torque value for each participant was used as their MVC torque in further analyses. During all experiments, the participants were instructed to remain in the position as seen in Fig. 1, and no knee or hip flexion or extension occurred, as observed visually by the investigator.

Participants then performed isometric ramp contractions up to a torque of 30% MVC at three different rates of force development: 2% MVC/s (slow), 10% MVC/s (medium) and 20% MVC/s (fast) guided by visual feedback. When the target torque was reached, participants were asked to hold the torque constant for several seconds (i.e. plateau region) before relaxing. Only the ramp data, i.e. from rest to the target torque, was used for this study, thus excluding the data from the brief plateau region. Prior to data collection, each participant practiced all three ramp rates until they were deemed to be competent by the experimenter. A period of rest was given between each contraction to avoid any possible fatigue. All ramps were administered in random order.

A schematic of the data collection setup and example signals from a representative subject are shown in Fig. 1. Torque signals were amplified 100 times (BK 1-5, Nobel Elektronik, Karlskoga, Sweden), low-pass filtered at 25 Hz and then analogue-to-digital converted at a sampling rate of 200 Hz (Micro 1401-3, Spike2 software, Cambridge Electronic Design, Cambridge, UK). Both the target torque and the filtered torque output were displayed on a monitor in front of the participants to provide visual feedback of their performance in matching the target ramp.

B-mode ultrasound videos of the MG were recorded (Telemed Echoblaster 128 CEXT system, Vilnius, Lithuania) at 80 Hz using a 60 mm linear transducer (Telemed, Vilnius, Lithuania) in a custom foam casing, placed over the thickest part of the MG muscle belly and in line with the longitudinal direction of the muscle fascicles. The transducer was then securely strapped to the leg using an elastic bandage (not shown in Fig. 1 for clarity) to prevent any out of plane movement.

### Protocol and data collection—experiment 2

**Fig. 2:**
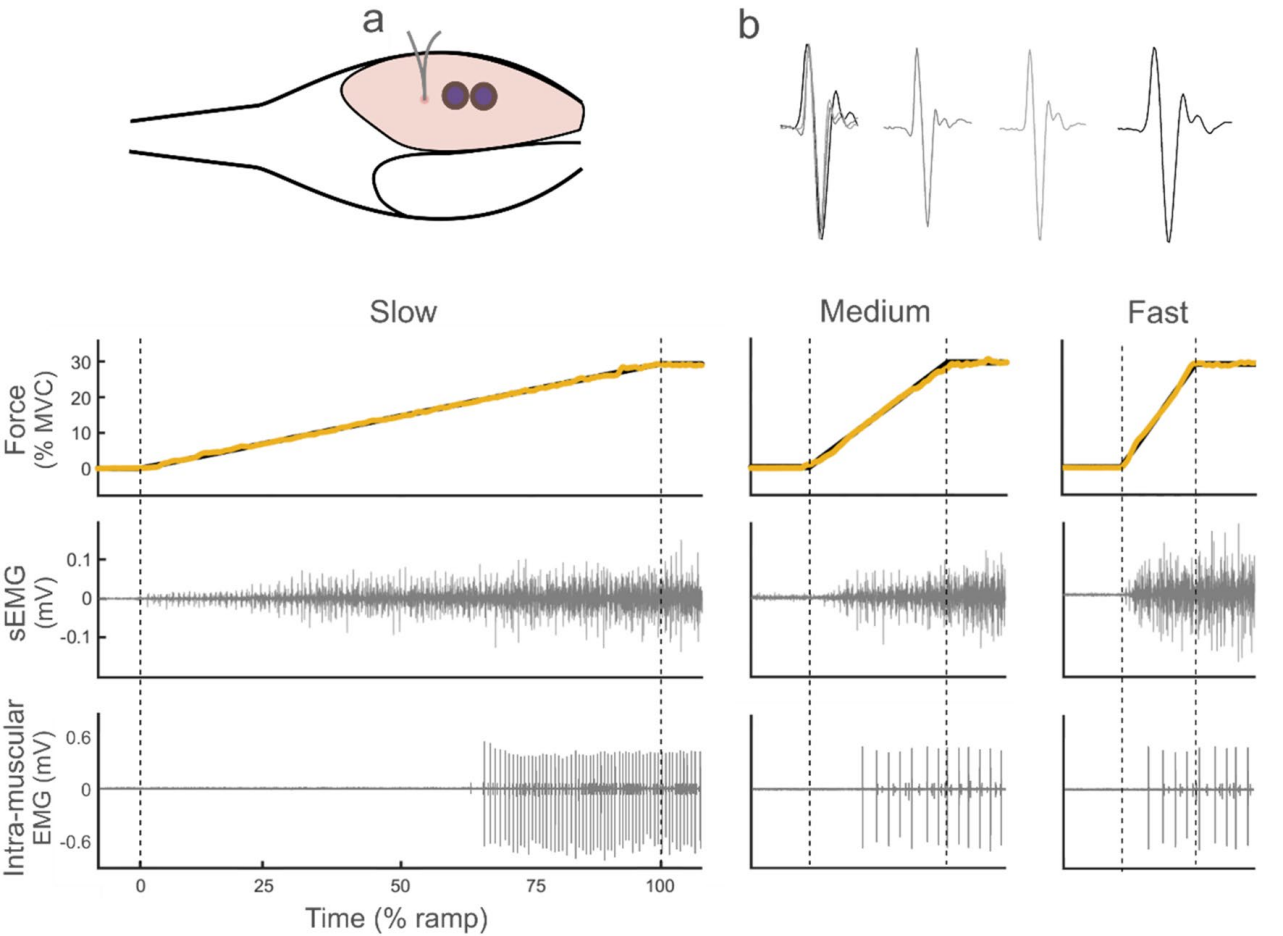
Top panel: a zoomed-in top view for experiment 2, using the same setup as shown in Fig. 1. Single motor unit action potentials were obtained from intramuscular bipolar fine-wire electrodes and surface EMG (EMGs) from MG (blue) and tibialis anterior (not shown) were recorded (**a**). Examples of single motor unit action potentials are shown for each condition (b), first overlain (left), then for the slow, medium and fast conditions separately. Bottom panel: example data from one participant for all three conditions (slow, medium, fast). Target torque (black) and the participant’s torque (orange) on top. Medial gastrocnemius EMGs root mean square and the action potential spikes of a single motor unit below. 1-s before and after each ramp is shown for all data except for the motor unit action potential spikes as these depend on when the motor unit was recruited. The vertical dashed lines mark the start and end of each ramp

With the relatively small size of the MG muscle, there is insufficient space for the ultrasound transducer, the surface EMG electrodes and indwelling fine-wire electrodes to be used together. Particularly because we needed to retract the wires of the indwelling electrodes several times (by 1 mm) to make measurements in slightly different locations in the muscle. Therefore, a separate experiment was conducted with the same setup as experiment 1 (Fig. 2) with EMG measurements taken instead of ultrasound. Participants went through the same protocol for determining their MVC torque. The target torque for this experiment was then defined as the highest torque up to which individual action potentials of at least one motor unit could be visually discriminated (see motor unit methods below; mean target torque: 26 ± 10% MVC, ranging between 15 and 50% MVC). Determining an individual target torque was necessary because the torque at which a motor unit could clearly be identified for several discharges can vary substantially between individuals. Then, each participant performed isometric ramp contractions to their individual target torque at the same three rates as in experiment 1 (2% MVC/s (slow), 10% MVC/s (medium) and 20% MVC/s (fast)).

Single motor units were recorded using a pair of fine-wire electrodes. A sterile needle (0.6 × 32 mm; BD Technologies, NJ, USA) containing a pair of Teflon coated, stainless steel wires (140 μm, half hard; A-M Systems, WA, USA) was inserted into the MG muscle under ultrasound guidance (Sonix MDP Ultrasonix, British Columbia, Canada). The needle was inserted to a depth of approximately 20 mm. The ends of the wires were fabricated with exposed 1 mm hooks, separated by 1 mm that allowed the wires to remain in the muscle when the needle was carefully retracted prior to data collection. During the experiment, the wires were retracted by 1 mm several times to make measurements in slightly different locations in the muscle, and thus increasing the motor unit yield. Intramuscular signals were amplified 1000 times (NL125, 126, Neurolog NL900D; Digitimer, UK) and band-pass filtered between 500 Hz and 5 kHz, before analogue-to-digital conversion at 20 kHz using the same hardware and software as used for the torque and EMGs data.

Bipolar surface EMG (EMGs) electrodes were placed over the MG and tibialis anterior muscle belly with an inter-electrode centre-to-centre distance of 20 mm. A reference electrode was placed on the lateral condyle of the femur. EMGs signals were amplified 1000 times and band-pass filtered between 10 and 500 Hz (NL 134, Neurolog NL900D; Digitimer, UK) before being analogue-to-digital converted at a sampling rate of 2 kHz using the same hardware and software as used for the torque data.

### Data processing and analyses

The filtered torque data from both experiments were exported to Matlab (R2018b, The Mathworks, MA, USA) and used to define the start and end of each ramp. An increase in torque greater than two times the standard deviation of the signal at rest was used to define the start, with the end defined as the time when the torque reached the target torque. The root mean square (RMS) error between the torque data and the target data was calculated to determine how closely the participants followed the target ramp. To quantify torque fluctuations, the linear trend, calculated as the best-fit straight line, was removed from the torque data and the standard deviation calculated.

Ultrasound videos were processed offline using a semi-automatic algorithm implemented in Matlab, using UltraTrack software (Farris and Lichtwark 2016). The orientation of the mean MG fascicles was defined on the first image of the video, ranging from the superficial to the deep aponeurosis along the echoes visible between the fascicles (Fig. 1). The length of the fascicle was then calculated relative to the known image depth. An affine-flow algorithm within the software was then used to track the length change of the fascicles in a frame by frame, iterative process (Cronin et al. 2011; Aeles et al. 2017). Each frame was visually inspected and corrected where needed. The change in fascicle length across the contraction period was calculated by subtracting the fascicle length at the start of the ramp from the fascicle length at the end of the ramp. The average fascicle shortening velocity for each ramp was calculated as the derivative of the length change with respect to time. Fascicle velocities are reported in absolute terms (mm/s) as well as relative to resting lengths (*L*_R_/s).

Single motor unit action potentials were discriminated from the intramuscular electrodes using Spike2 software. Action potentials were classified based on shape and amplitude using a semi-automatic approach. Visual examination of each action potential confirmed that they were classified to the correct motor unit. Only motor units of which action potentials could be clearly discriminated between recruitment and the end of the ramp for all three ramp rates were used for further analyses, which significantly reduces the total number of motor units available for analysis, but was a requirement for testing our hypothesis. All action potentials were verified on two separate occasions. The recruitment threshold of each motor unit was determined in Matlab by moving a 500-ms window forward in steps of 1 ms until the inter-spike interval coefficient of variation within the window was ≤ 0.5 (Moritz et al. 2005). Motor units with recruitment thresholds beyond the end of the ramp were discarded. Inter-spike intervals were used to calculate the instantaneous firing frequency of each motor unit. The firing frequency at recruitment was defined as the initial firing frequency, using the first two action potentials and the frequency at the end of the ramp was defined as the final firing frequency, using the last two action potentials. The number of action potentials during the ramp was also calculated for each motor unit. While all motor units were used for assessment of the recruitment threshold and initial and final discharge frequency, only motor units with five or more action potentials during the ramp were used to calculate the mean firing frequency. For the MG surface EMG recording, the root mean square (RMS) amplitude of the EMGs over the entire ramp was calculated.

### Statistical analysis

A comparison in torque matching parameters between the two experiments was performed using two-way ANOVAs. Repeated measures ANOVAs were performed with *P* values corrected using the Greenhouse–Geisser method to compare between rates of force development for the total amount of fascicle shortening and the average fascicle velocity as well as the RMS amplitudes for the surface EMGs. To compare between conditions for the motor unit recruitment threshold, initial discharge frequency, final discharge frequency and mean discharge frequency, a repeated measures nested linear mixed model was used, with motor units nested in participants (Tenan et al. 2014) and the rate of force development as the fixed effect and the participant and the respective motor unit as random effects. The model equation had the form:

$$y \sim 1+\mathrm{Rate}+\left(1|\mathrm{Participant}:MUnr\right)+(1|\mathrm{Participant})$$

With *y* denoting one of the input parameters (e.g. recruitment thresholds), *Rate* representing the categorical rate of force development (e.g. slow), *Participant* represents the participant identification number and *MUnr* the motor unit identification number for each participant. The Satterthwaite method for obtaining degrees of freedom was then used and the rate of force developments were compared with *F* tests. Tukey’s method was used for pairwise comparison for significant main effects. We used repeated measures correlations (Bland and Altman 1995; Marusich and Bakdash 2021) to test the relation between the change in recruitment threshold and change in mean discharge frequency between each of two conditions. Correlation outcomes were interpreted according to the qualitative scale used in Koo and Li (2017). Significance was defined as *P* ≤ 0.05.

## Results

The RMS error and standard deviation of the plantar flexion torque increased with increasing ramp rate and was significantly different between all three conditions for both sets of experiments (*P* < 0.01). However, the low errors show that the participants were able to match the target ramps very well, and there was no statistical difference in the RMS error nor the standard deviation between the two experiments (Table 1).

**Table 1:** Torque matching parameters

	Ramp rate	Experiment 1 (mean ± SD)	Experiment 2 (mean ± SD)	*P* value
RMS error (% MVC)	Slow	0.31 ± 0.09	0.34 ± 0.09	0.12
	Medium	0.93 ± 0.36	0.71 ± 0.17	
	Fast	1.59 ± 0.72	1.33 ± 0.37	
SD (% MVC)	Slow	0.31 ± 0.06	0.38 ± 0.11	0.90
	Medium	0.70 ± 0.34	0.68 ± 0.21	
	Fast	0.96 ± 0.28	0.90 ± 0.52	

Values are mean ± standard deviations for both the root mean square (RMS) error and the standard deviation (SD). *P* values are from the statistical comparison between both data collections

**Fig. 3:**
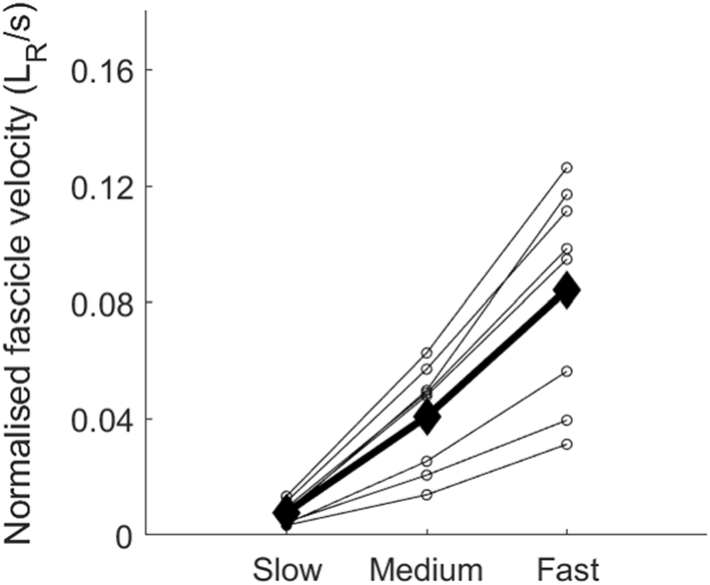
Medial gastrocnemius normalised fascicle velocities for the slow, medium, and fast conditions (experiment 1). Small circles connected by the dotted lines are the individual values for each participant (*N* = 8) and filled diamonds and thick black lines are the mean values for each condition

There was no significant change in the total amount of shortening of the MG fascicles at peak force across the three rate of force development conditions (5.7 ± 2.9 mm, 5.9 ± 2.8 mm, 6.1 ± 2.8 mm, slow, medium and fast, respectively). However, a significant main effect of rate of force development on the shortening velocity of the MG fascicles was found (*P* < 0.01). The shortening velocity of the fascicles increased from 0.4 ± 0.2 mm/s to 2.0 ± 0.9 mm/s to 4.1 ± 1.9 mm/s (0.01 ± 0.00 *L*_R_/s to 0.04 ± 0.02 *L*_R_/s to 0.08 ± 0.04 *L*_R_/s), *P* < 0.02, between the slow and medium, and medium and fast conditions, respectively (Fig. 3). Importantly, and as expected, the shortening velocities increased with increasing rate of force development in each participant. This invariant behaviour confirmed the premise we set for experiment 2, allowing us to then study the effect this increase in fascicle shortening velocity has on motor unit discharge behaviour and whole muscle activation.

**Fig. 4:**
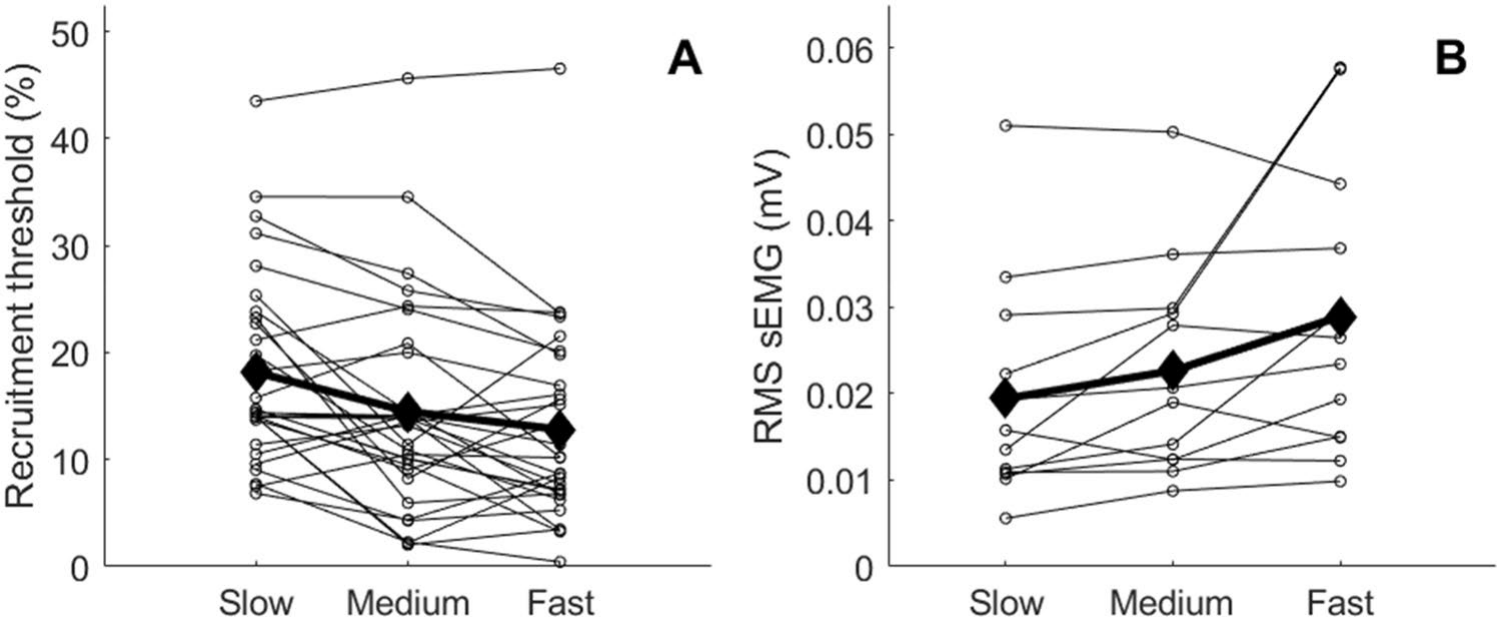
Medial gastrocnemius motor unit recruitment thresholds (**A**) and root mean square (RMS) amplitude of the surface EMG (**B**) for the slow, medium, and fast conditions (experiment 2). Small circles connected by the dotted lines are the individual values for each participant (*N* = 30 motor units, *N* = 12 measurements from 9 participants for **A** and **B,** respectively) and filled diamonds and thick black lines are mean values per conditions. The values in **B** should not be compared across individuals

Between two and five motor units were identified per participant. In total, this results in 30 motor units that were discriminated and analysed. The recruitment threshold of these motor units was significantly different between the conditions. The multiple comparisons test revealed that the recruitment threshold was lower in the fast (12.8 ± 9.2% MVC, *P* < 0.01) and medium (14.5 ± 9.9% MVC, *P* = 0.01) conditions compared to the slow condition (18.2 ± 8.9% MVC), but not different between the medium and fast conditions (Fig. 4). The initial discharge frequency was lower in the slow (5.8 ± 3.1 Hz) compared to the fast (6.7 ± 1.4 Hz) condition (*P* < 0.01), but not between the medium (6.4 ± 2.4 Hz) and slow or medium and fast conditions. The final discharge frequencies (9.5 ± 3.8 Hz, 9.2 ± 2.7 Hz, 9.5 ± 3.3 Hz for slow, medium and fast, respectively) of all 30 motor units were not significantly different between the ramp rates. Of these 30 motor units, 11 discharged fewer than the required 5 action potentials during the ramp and were therefore not used for determining mean discharge frequencies. The remaining 19 motor units discharged an average of 38 ± 19, 15 ± 5, 9 ± 3 action potentials for the slow, medium, and fast conditions, respectively. The mean discharge frequency of those units was not significantly different between the three ramp rates (9.6 ± 2.3 Hz, 9.5 ± 1.5 Hz and 9.7 ± 2.3 Hz, slow, medium and fast, respectively). A negative correlation was found between the change in recruitment threshold between the slow and fast and the corresponding change in mean discharge frequency between the slow and fast (*r*^2^ = 0.58, *P* = 0.02) and between the change in recruitment threshold between the medium and fast and the corresponding change in mean discharge frequency between the medium and fast (*r*^2^ = 0.66 *P* < 0.01) conditions. No correlation was found between the slow and medium (*r*^2^ = 0.027, *P* = 0.667) conditions.

The RMS amplitude of MG EMGs was significantly different between the conditions (*P* = *0.03)*. The multiple comparisons test revealed that the RMS amplitude was greater for the fast compared to slow ramps (mean RMS: 0.029 ± 0.017 mV versus 0.019 ± 0.013 mV, respectively, *P* = 0.05) but was not different between the medium ramp (0.023 ± 0.012 mV) and the other two conditions (Fig. 4). The RMS amplitude of tibialis anterior EMGs was not different between the conditions (mean RMS: 0.006 ± 0.003 mV, 0.006 ± 0.003 mV, 0.007 ± 0.004 mV for the slow, medium and fast conditions, respectively).

## Discussion

In this study, we examined how muscle fascicle shortening velocity changes with small increases in rate of force development and demonstrate the subsequent change in motor unit discharge behaviour responsible for generating force under the same rate of force development conditions. We show that with increasing rate of force development, there is a significant increase in muscle fascicle shortening velocity, which must result in a drop in the force-generating capacity of the muscle according to the force–velocity relationship. We show that concomitant with the increased shortening velocity, under the same contraction conditions, there was an increase in neural drive (i.e. lower motor unit recruitment thresholds with no changes in discharge frequencies), relative to force output. Furthermore, a higher overall RMS amplitude of the MG surface EMG was found. We suggest that the increased neural drive is a requirement to produce the force required given the reduced force capacity of muscle fascicles and that this effect is likely to be greater for muscles with higher series compliance.

### Muscle fascicle dynamics

**Fig. 5:**
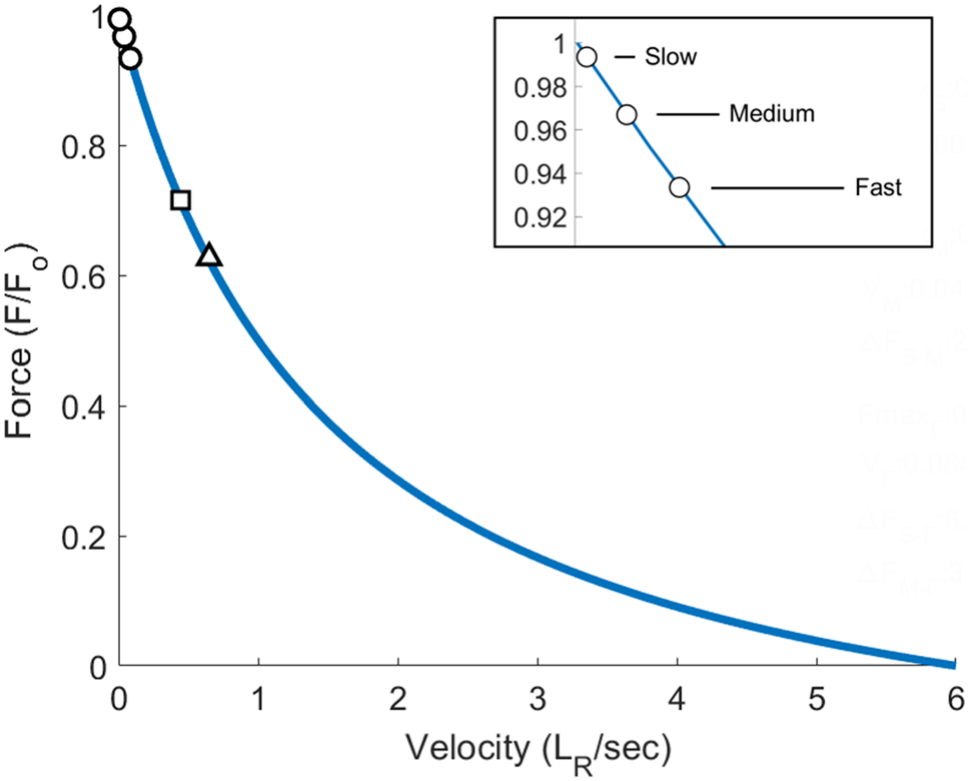
Theoretical force–velocity curve based on formulas from Woledge et al. (1985), with normalised force (*F*_0_). Note that this parabolic relationship holds for muscle force–velocity properties but may differ when joint dynamics are considered (Bobbert 2012). The maximal velocity of the muscle was set at 6 *L*_R_/s based on Hager et al. (2018). The open circles represent the average shortening velocities of the muscle fascicles in this study, resulting in a maximal force capacity drop of 0.7%, 3.3%, and 6.6% for the slow, medium and fast condition, respectively. An enlarged view of this section of the curve is shown in the box in the figure. As a reference, the open square and triangle represent the average shortening velocities during walking and running, respectively (Farris and Sawicki 2012). Note that the fascicles during walking and running act mostly isometrically due to the compliance of the tendon. However, the fascicle velocities may be constrained differently during the different tasks. Maximum shortening velocity reported by Hager et al. (2018) is only an estimate and could be affected by differences in knee positions in our study compared to Hager et al. (2018). Regardless of these limitations, there is a significant drop in force-generating capacity of the muscle due to the increased shortening velocity with increases in rate of force development. We estimate that the actual drop in force during functional tasks, such as walking and running, is much greater than estimated for the isometric task in our experiments

The setup used in our experimental protocol required an isometric plantarflexion contraction for which the whole muscle–tendon unit length changes minimally. However, due to the in-series compliance of tendons, muscle fascicles significantly shorten even during such isometric contractions (Griffiths 1984, 1987). Our results support our hypothesis that shortening velocity would increase with rate of force development as a result of the tendinous compliance. When muscle fascicles shorten at velocities greater than zero, their maximal force-generating capacity is reduced as per the force–velocity relationship (Hill 1938), even during isometric contractions. The fascicle shortening velocities in the current study are estimated to result in a theoretical drop in maximal force capacity of 0.7 ± 0.3%, 3.3 ± 1.4%, 6.6 ± 2.8% for the slow, medium and fast condition, respectively (*P* < 0.01; Fig. 5; see supplementary information for calculations). However, the shortening velocities in the MG are typically higher during daily tasks, e.g. 0.4—0.6 *L*_R_/s in walking and running (Farris and Sawicki 2012), which theoretically results in a 30–40% drop in maximal force-generating capacity during those tasks. Therefore, added neural drive during rapid force development is a likely requirement during many daily activities, to compensate for the decline in force-generating capacity induced by even greater fascicle shortening velocities compared to the velocities observed in our experiment. This is supported by the large changes in recruitment threshold with increasing contraction velocities in studies that investigated relatively slow versus very fast movements (Budingen and Freund 1976; Desmedt and Godaux 1977a) as well as by the increases in amplitude of surface EMG recordings, which further suggests additional motor unit recruitment (Bigland and Lippold 1954; Sale 1987; MacIntosh et al. 1999; Farina et al. 2004).

### Motor unit behaviour

We did not observe a significant difference in recruitment threshold between the medium and fast condition, despite a predicted similar drop in maximal force capacity compared to the slow to medium condition. At these relatively low velocities and forces, it is likely that a low number of motor units are active and that not all are required to lower their recruitment thresholds. It is thus probable that changes occurred in the recruitment threshold of other motor units or that new motor units were recruited that were not detected by the highly selective fine-wire electrodes. This was however not supported by the surface EMG amplitude between the slow and medium, and medium and fast conditions. We believe that the effect would be more sizeable with significantly higher rates of force development. For example, early studies using fine-wire techniques did not find changes in recruitment threshold when increasing the rate of force development at low velocities but did find changes at higher velocities (Budingen and Freund 1976; Desmedt and Godaux 1977a, b). Alternatively, the reduced compliance in the different muscles examined (e.g. extensor indicis in Budingen and Freund 1976 and tibialis anterior in Desmedt and Godaux 1977a, b) will also reduce the differences in shortening velocity between conditions and hence the effect is likely to be less clear in muscles with low series compliance. However, a recruitment strategy is supported by the significant increase in whole muscle activation (surface EMG amplitude) between the slow and fast conditions in the current study, although surface EMG amplitude is both influenced by recruitment and rate coding.

Despite observing no changes in motor unit discharge frequencies, we did find significant negative correlations between the change in recruitment threshold and the change in mean discharge frequencies for the slow to fast and medium to fast conditions. It is plausible that an earlier recruitment of the motor units, plus an increase in their discharge frequencies, would generate too much force, which would cause the participants to overshoot the force target. Studies that have compared relatively slow movements to ballistic (very fast) movements have reported increases in motor unit discharge frequencies (Grimby and Hannerz 1977; Desmedt and Godaux 1977a, b, 1979; Duchateaux and Baudry 2014). However, for the relatively low rates of force development examined in our study, lowering recruitment thresholds seemed to dominate over changes in firing frequency, although we did find lower initial firing frequencies in the slow compared to the fast condition.

### Pairing muscle dynamics with motor unit behaviour

While competing theories propose a range of reasons why recruitment threshold decreases with increasing rate of force development, the changes in muscle force-generating capacity due to increases in fascicle shortening velocity represents the most plausible reason and to date has not been fully discussed in this context. There may also be a shift in muscle recruitment from muscles with predominantly slow-twitch to muscles with predominantly fast-twitch fibres, yet this seems unlikely to be significant in the small changes in rate of force development in our experiments. The change in discharge behaviour for a change in rate of force development may also differ between muscles (Desmedt and Godaux 1978). While this may affect the magnitude of changes in neural drive for a given change in rate of force development between muscles, when the shortening velocities of the muscle fascicles increase, the drop in force-generating capacity should result in an increase in neural drive to maintain the target force output. We also did not find any changes between any of the conditions in an important plantar flexion antagonist, tibialis anterior, ruling out an increase in neural drive to the MG to compensate for increased co-contraction of its antagonist. Another plausible contributing factor is the greater weight of the electromechanical delay in the muscle at greater rates of force development. However, the actual delay in the MG has been shown to be rather short, i.e. 12 ms (Nordez et al. 2009), and we estimate the effect it has on motor unit recruitment thresholds to be of minimal impact in the conditions we tested. In our study, the visual stimulus that was provided to the participants constrained the rate of force development. This stimulus informs the nervous system of the task requirements to achieve the task, in this case increasing force development at a prescribed rate. While our results show that increasing the rate of force development resulted in both greater fascicle shortening velocities as well as an increase in the neural drive, the feedforward control (with potential feedback from visual stimulus) requires that the participants drive motor units to generate the required force, which subsequently influences the fascicle shortening velocity. Therefore, fascicle shortening velocity is unlikely to dictate the required motor unit recruitment. Instead, our results highlight the need for the increase in neural drive, given that the fascicle dynamics cause a drop in the force-generating capacity of the muscle. If the neural drive was not increased, then the drop in force-generating capacity would result in a force output that fell short of the target. In conclusion, while we cannot extract cause and effect from these experiments, our findings do provide an explanation for the need to increase the neural drive observed in previous studies that remained unexplained. Such effects of velocity are commonly inherently handled in muscle models of contraction (Zajac 1989; Millard et al. 2013), but have been somewhat ignored when considering motor unit recruitment in isometric contraction conditions.

### Considerations

While we only tested the MG muscle, other muscles, such as the lateral gastrocnemius and soleus, also contribute to the plantar flexion torque required to perform the task in our experiments. It is possible that the behaviour of the slower yet forceful soleus muscle may have affected the behaviour of the MG. Further studies will need to confirm the validity of the explanation for the increase in neural drive with increases in rate of force development for other muscles. Ideally, the two separate experiments performed here would be performed in the same session on the same experiments. However, the requirement to securely strap the ultrasound transducer, interferes with the proper use of fine-wire electrodes. A third limitation of this study is that the experiments only covered relatively slow rates of force development. Because of the parabolic nature of the force–velocity relationship, the effect will be largest at lower rates of shortening but greater differences between the conditions may have induced larger changes in recruitment threshold and potentially also significant changes in mean discharge frequency. However, the fact that our results support the proposed theory even under these relatively small changes in rate of force development, strengthens our assertion that it is based on the inherent characteristics of fascicle dynamics during contraction of skeletal muscle. The task of matching the rate of force development presents an interesting challenge for the nervous system, which must integrate the feedback from muscles, tendon and visual feedback in an instance to match the right amount of neural drive for the required force output.

## Supplementary Information

Below is the link to the electronic supplementary material.

Supplementary file1 (DOCX 27 kb)

## Data Availability

The authors have made the data available: https://figshare.com/s/8faf18da1fc72c8fb20a
