# Supplementary material for: The effect of small changes in rate of force development on muscle fascicle velocity and motor unit discharge behaviour
Source: Eur J Appl Physiol. 2022 Feb 10;122(4):1035–44. doi: 10.1007/s00421-022-04905-7 (PMC8926959; doi:10.1007/s00421-022-04905-7)
Supplement: Supplementary file 1 — Supplementary file1 (DOCX 27 kb) [file 421_2022_4905_MOESM1_ESM.docx]

**Supplementary information**

A theoretical change in force due to change in fascicle length (velocity) was estimated using a theoretical force-velocity curve using the formula described by Woledge (1985):

$$P^{'}={(1-V^{'})}/{(1+V^{'}G)}$$

where $P^{'}$ is the force relative to the maximal force ($P_{0}$), $V^{'}$ is the fascicle velocity relative to the maximal velocity ($V_{0}$), and $G$ is (${P_{0}}/a$) with constant $a$ equal to 0.25 (Hill 1938). The maximal MG fascicle shortening velocity was set to six lengths ${{per second (6L}_{0}}/{s)}$ (Hager et al., 2018).
